# Using the consolidated Framework for Implementation Research to integrate innovation recipients’ perspectives into the implementation of a digital version of the spinal cord injury health maintenance tool: a qualitative analysis

**DOI:** 10.1186/s12913-024-10847-x

**Published:** 2024-03-28

**Authors:** John A Bourke, K. Anne Sinnott Jerram, Mohit Arora, Ashley Craig, James W Middleton

**Affiliations:** 1 John Walsh Centre for Rehabilitation Research, Northern Sydney Local Health District, St Leonards, NSW Australia; 2 The Kolling Institute, Faculty of Medicine and Health, The University of Sydney, Sydney, NSW Australia; 3 Burwood Academy Trust, Burwood Hospital, Christchurch, New Zealand; 4 Royal Rehab, Ryde, NSW Australia; 5 State Spinal Cord Injury Service, NSW Agency for Clinical Innovation, St Leonards, NSW Australia

**Keywords:** Spinal Cord injury, Self-management, Innovation recipients, Secondary health conditions, Primary health care, Evidence-based innovations, Actionable findings, Consolidated Framework for implementation research, CFIR

## Abstract

**Background:**

Despite advances in managing secondary health complications after spinal cord injury (SCI), challenges remain in developing targeted community health strategies. In response, the SCI Health Maintenance Tool (SCI-HMT) was developed between 2018 and 2023 in NSW, Australia to support people with SCI and their general practitioners (GPs) to promote better community self-management. Successful implementation of innovations such as the SCI-HMT are determined by a range of contextual factors, including the perspectives of the innovation recipients for whom the innovation is intended to benefit, who are rarely included in the implementation process. During the digitizing of the booklet version of the SCI-HMT into a website and App, we used the Consolidated Framework for Implementation Research (CFIR) as a tool to guide collection and analysis of qualitative data from a range of innovation recipients to promote equity and to inform actionable findings designed to improve the implementation of the SCI-HMT.

**Methods:**

Data from twenty-three innovation recipients in the development phase of the SCI-HMT were coded to the five CFIR domains to inform a semi-structured interview guide. This interview guide was used to prospectively explore the barriers and facilitators to planned implementation of the digital SCI-HMT with six health professionals and four people with SCI. A team including researchers and innovation recipients then interpreted these data to produce a reflective statement matched to each domain. Each reflective statement prefaced an actionable finding, defined as alterations that can be made to a program to improve its adoption into practice.

**Results:**

Five reflective statements synthesizing all participant data and linked to an actionable finding to improve the implementation plan were created. Using the CFIR to guide our research emphasized how partnership is the key theme connecting all implementation facilitators, for example ensuring that the tone, scope, content and presentation of the SCI-HMT balanced the needs of innovation recipients alongside the provision of evidence-based clinical information.

**Conclusions:**

Understanding recipient perspectives is an essential contextual factor to consider when developing implementation strategies for healthcare innovations. The revised CFIR provided an effective, systematic method to understand, integrate and value recipient perspectives in the development of an implementation strategy for the SCI-HMT.

**Trial registration:**

N/A.

**Supplementary Information:**

The online version contains supplementary material available at 10.1186/s12913-024-10847-x.

## Background

Injury to the spinal cord can occur through traumatic causes (e.g., falls or motor vehicle accidents) or from non-traumatic disease or disorder (e.g., tumours or infections) [1]. The onset of a spinal cord injury (SCI) is often sudden, yet the consequences are lifelong. The impact of a SCI is devastating, with effects on sensory and motor function, bladder and bowel function, sexual function, level of independence, community participation and quality of life [2]. In order to maintain good health, wellbeing and productivity in society, people with SCI must develop self-management skills and behaviours to manage their newly acquired chronic health condition [3]. Given the increasing emphasis on primary health care and community management of chronic health conditions, like SCI, there is a growing responsibility on all parties to promote good health practices and minimize the risks of common health complications in their communities.

To address this need, the Spinal Cord Injury Health Maintenance Tool (SCI-HMT) was co-designed between 2018 and 2023 with people living with SCI and their General Practitioners (GPs) in NSW, Australia [4] The aim of the SCI-HMT is to support self-management of the most common and arguably avoidable potentially life-threatening complications associated with SCI, such as mental health crises, autonomic dysreflexia, kidney infections and pressure injuries. The SCI-HMT provides comprehensible information with resources about the six highest priority health areas related to SCI (as indicated by people with SCI and GPs) and was developed over two phases. Phase 1 focused on developing a booklet version and Phase 2 focused on digitizing this content into a website and smartphone app [4, 5].

Enabling the successful implementation of evidence-based innovations such as the SCI-HMT is inevitably influenced by contextual factors: those dynamic and diverse array of forces within real-world settings working for or against implementation efforts [6]. Contextual factors often include background environmental elements in which an intervention is situated, for example (but not limited to) demographics, clinical environments, organisational culture, legislation, and cultural norms [7]. Understanding the wider context is necessary to identify and potentially mitigate various challenges to the successful implementation of those innovations. Such work is the focus of determinant frameworks, which focus on categorising or classing groups of contextual determinants that are thought to predict or demonstrate an effect on implementation effectiveness to better understand factors that might influence implementation outcomes [8].

One of the most highly cited determinant frameworks is the Consolidated Framework for Implementation Research (CFIR) [9], which is often posited as an ideal framework for pre-implementation preparation. Originally published in 2009, the CFIR has recently been subject to an update by its original authors, which included a literature review, survey of users, and the creation of an outcome addendum [10, 11]. A key contribution from this revision was the need for a greater focus on the place of innovation recipients, defined as the constituency for whom the innovation is being designed to benefit; for example, patients receiving treatment, students receiving a learning activity. Traditionally, innovation recipients are rarely positioned as key decision-makers or innovation implementers [8], and as a consequence, have not often been included in the application of research using frameworks, such as the CFIR [11].

Such power imbalances within the intersection of healthcare and research, particularly between those receiving and delivering such services and those designing such services, have been widely reported [12, 13]. There are concerted efforts within health service development, health research and health research funding, to rectify this power imbalance [14, 15]. Importantly, such efforts to promote increased equitable population impact are now being explicitly discussed within the implementation science literature. For example, Damschroder et al. [11] has recently argued for researchers to use the CFIR to collect data from innovation recipients, and that, ultimately, “equitable population impact is only possible when recipients are integrally involved in implementation and all key constituencies share power and make decisions together” (p. 7). Indeed, increased equity between key constituencies and partnering with innovation recipients promotes the likelihood of sustainable adoption of an innovation [4, 12, 14].

There is a paucity of work using the updated CFIR to include and understand innovation recipients’ perspectives. To address this gap, this paper reports on a process of using the CFIR to guide the collection of qualitative data from a range of innovation recipients within a wider co-design mixed methods study examining the development and implementation of SCI-HMT. The innovation recipients in our research are people living with SCI and GPs. Guided by the CFIR domains (shown in the supplementary material), we used reflexive thematic analysis [16]to summarize data into reflective summaries, which served to inform actionable findings designed to improve implementation of the SCI-HMT.

## Methods

### Procedure

**Fig. 1:**
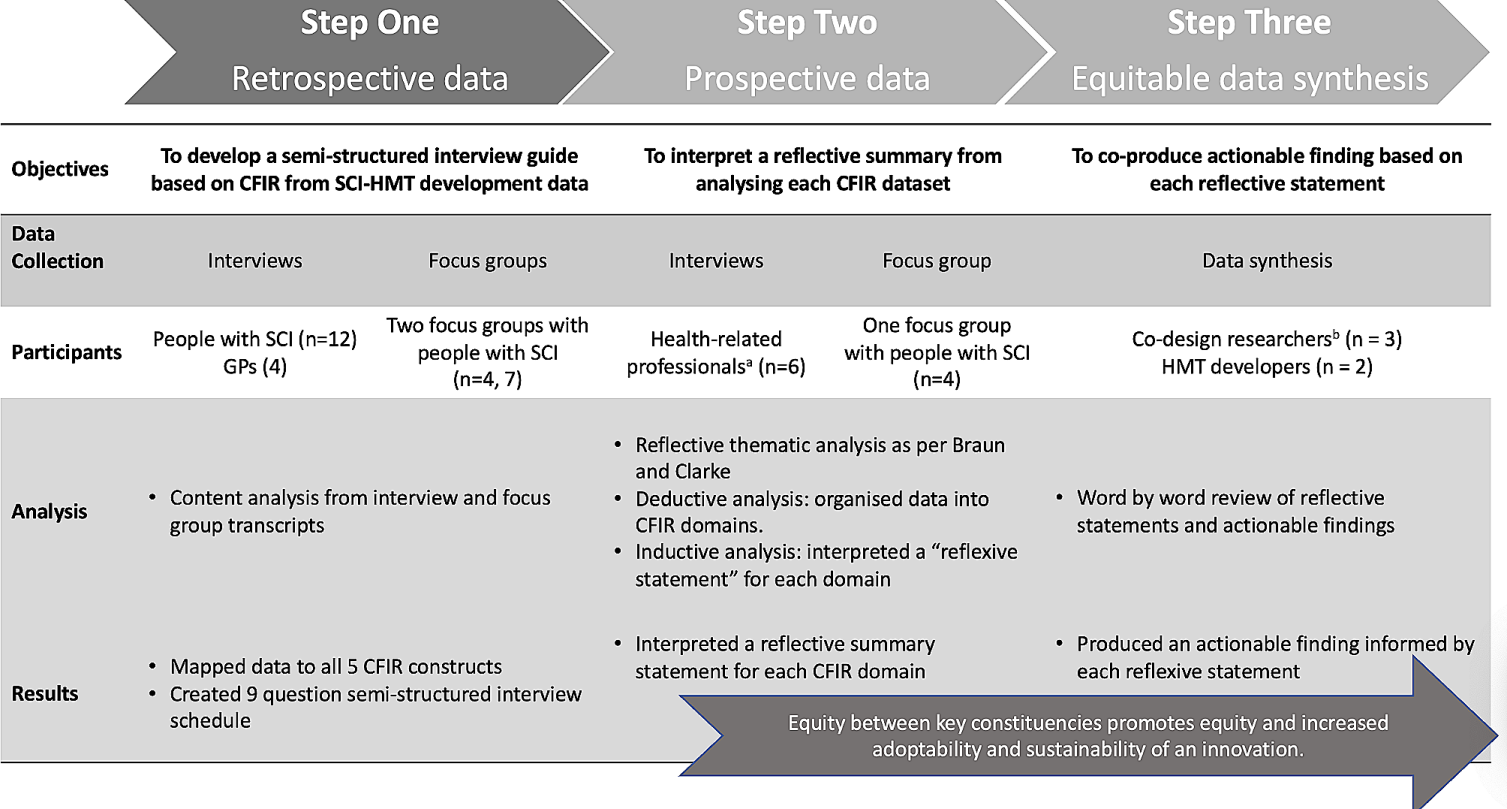
Procedure of synthesising datasets to inform reflective statements and actionable findings. ^a^Two health professionals had a SCI (one being JAB); ^b^Two co-design researchers had a SCI (one being JAB)

The procedure for this research is multi-stepped and is summarized in Fig. 1. First, we mapped retrospective qualitative data collected during the development of the SCI-HMT [4] against the five domains of the CFIR in order to create a semi-structured interview guide (Step 1). Then, we used this interview guide to collect prospective data from health professionals and people with SCI during the development of the digital version of the SCI-HMT (Step 2) to identify implementation barriers and facilitators. This enabled us to interpret a reflective summary statement for each CFIR domain. Lastly, we developed an actionable finding for each domain summary. The first (RESP/18/212) and second phase (2019/ETH13961) of the project received ethical approval from The Northern Sydney Local Health District Human Research Ethics Committee. The reporting of this study was conducted in line with the consolidated Criteria for Reporting Qualitative Research (COREQ) guidelines [17]. All methods were performed in accordance with the relevant guidelines and regulations.

### Step one: retrospective data collection and analysis

We began by retrospectively analyzing the data set (interview and focus group transcripts) from the previously reported qualitative study from the development phase of the SCI-HMT [4]. This analysis was undertaken by two team members (KASJ and MA). KASJ has a background in co-design research. Transcript data were uploaded into NVivo software (Version 12: QSR International Pty Ltd) and a directed content analysis approach [18] was applied to analyze categorized data a priori according to the original 2009 CFIR domains (intervention characteristics, outer setting, inner setting, characteristics of individuals, and process of implementation) described by Damschroder et al. [9]. This categorized data were summarized and informed the specific questions of a semi-structured interview guide. The final output of step one was an interview guide with context-specific questions arranged according to the CFIR domains (see supplementary file 1). The interview was tested with two people with SCI and one health professional.

### Step two: prospective data collection and analysis

In the second step, semi-structured interviews were conducted by KASJ (with MA as observer) with consenting healthcare professionals who had previously contributed to the development of the SCI-HMT. Healthcare professionals included GPs, Nurse Consultants, Specialist Physiotherapists, along with Health Researchers (one being JAB). In addition, a focus group was conducted with consenting individuals with SCI who had contributed to the SCI-HMT design and development phase. The interview schedule designed in step one above guided data collection in all interviews and the focus group.

The focus group and interviews were conducted online, audio recorded, transcribed verbatim and uploaded to NVivo software (Version 12: QSR International Pty Ltd). All data were subject to reflexive, inductive and deductive thematic analysis [16, 19] to better understand participants’ perspectives regarding the potential implementation of the SCI-HMT. First, one team member (KASJ) read transcripts and began a deductive analysis whereby data were organized into CFIR domains-specific dataset. Second, KASJ and JAB analyzed this domain-specific dataset to inductively interpret a reflective statement which served to summarise all participant responses to each domain. The final output of step two was a reflective summary statement for each CFIR domain.

### Step three: data synthesis

In the third step we aimed to co-create an actionable finding (defined as tangible alteration that can be made to a program, in this case the SCI-HMT [20]) based on each domain-specific reflective statement. To achieve this, three codesign researchers (KAS and JAB with one person with SCI from Step 2 (deidentified)) focused on operationalising each reflective statement into a recommended modification for the digital version of the SCI-HMT. This was an iterative process guided by the specific CFIR domain and construct definitions, which we deemed salient and relevant to each reflective statement (see Table 2 for example). Data synthesis involved line by line analysis, group discussion, and repeated refinement of actionable findings. A draft synthesis was shared with SCI-HMT developers (JWM and MA) and refinement continued until consensus was agreed on. The final outputs of step three were an actionable finding related to each reflective statement for each CFIR domain.

## Results

The characteristics of both the retrospective and prospective study participants are shown in Table 1. The retrospective data included data from a total of 23 people: 19 people with SCI and four GPs. Of the 19 people with SCI, 12 participated in semi-structured interviews, seven participated in the first focus group, and four returned to the second focus group. In step 2, four people with SCI participated in a focus group and six healthcare professionals participated in one-on-one semi-structured interviews. Two of the healthcare professionals (a GP and a registrar) had lived experience of SCI, as did one researcher (JAB). All interviews and focus groups were conducted either online or in-person and ranged in length between 60 and 120 min.

**Table 1:** Descriptive summary of retrospective and prospective study participants

**Retrospective data** (***n*** = **23**)		
Participant type	People with SCI	(*n* = 19)
	Age (years), mean (SD)	50.2 (14.9)
	Time since injury (years), mean (SD)	20.0 (15.6)
	Male, n (%)	10 (53)
	Female, n (%)	9 (47)
	Paraplegia, n (%)	7 (37)
	Tetraplegia, n (%)	12 (63)
	Complete injury, n (%)	9 (47)
	Incomplete injury, n (%)	10 (53)
	Remoteness	
	*Rural*, n (%)	6 (32)
	*Metro*, n (%)	13 (68)
Participant type	GPs	(*n* = 4)
	Male, n (%)	2 (50)
	Female, n (%)	2 (50)
	Remoteness, n (%)	
	*Rural*, n (%)	2 (50)
	*Metro*, n (%)	2 (50)
	Years of experience^a^, n (%)	
	*Less than 10 years*	1 (25)
	*10 years or more*	3 (75)
**Prospective data** (***n*** = ***10***)		
Participant type	People with SCI	(*n* = 4)
	Age (years), mean (SD)	35.5 (13.7)
	Time since injury (years), mean (SD)	6.0 (1.0)
	Male, n (%)	1 (25)
	Female, n (%)	3 (75)
	Paraplegia, n (%)	1 (25)
	Tetraplegia, n (%)	3 (75)
Participant type	Health-Related Professionals^a^	(*n* = 6)
	Male, n (%)	3 (50)
	Female, n (%)	3 (50)
	Years of experience^a^, n (%)	
	*Less than 10 years*	0 (0)
	*10 years or more*	6 (100)

In our overall synthesis, we actively interpreted five reflective statements based on the updated CFIR domain and construct definitions by Damschroder et al. [11]. Table 2 provides a summary of how we linked the updated CFIR domain and construct definitions to the reflective statements. We demonstrate this process of co-creation below, including illustrative quotes from participants. Importantly, we guide readers to the actionable findings related to each reflective statement in Table 2. Each actionable statement represents an alteration that can be made to a program to improve its adoption into practice.

**Table 2:** Summary of CFIR domain-Specific Reflective Statements Informing Actionable Findings

CFIR Domain^a^	CFIR construct and definition	Reflective summary synthesising innovation receipts feedback	Actionable findings
I. Innovation	*Adaptability (D)* The innovation can be modified, tailored, or refined to fit local context or needs.	Self-management was acknowledged as demanding and the SCI-HMT had to balance complexity with accessibility. Contact with healthcare for a person with SCI can be challenging. The SCI-HMT has the potential to facilitate improved communication with healthcare services.	The content of the SCI-HMT needs to mix expert clinical information with lived experience knowledge. The digitalized tool will aim to reduce the points of contact to save time and improves efficiency of communication and decision-making for people with SCI and their health care providers. Age does not necessarily present an e-literacy barrier.
II. Outer Setting	*Partnerships & Connections (D)* The Inner Setting is networked with external entities, including referral networks, academic affiliations, and professional organization networks.	Participants suggested that knowledge is power and that the SCI-HMT would have strong utility in post-acute rehabilitation services, as well as in primary care setting. SCI peer support networks were considered crucial to promoting the SCI-HMT. Champions from both SCI and primary health communities are needed to facilitate the tool’s integration and utilization.	The SCI-HMT should be promoted for use at all time points from post-acute rehabilitation onwards, and be circulated through SCI community groups, as well as primary and tertiary care centers to maximize uptake. Collaboration with College of GPs is recommended for recognition of the value of the six modules for CPD credentials.
III. Inner Setting	Recipient-Centeredness (D2) There are shared values, beliefs, and norms around caring, supporting, and addressing the needs and welfare of recipients.	Self-management of health and well-being is substantial and could potentially be resisted by people with SCI if they felt overwhelmed. While GP’s are time poor, support by GPs for self-management was considered essential. The SCI-HMT can help to synthesize self-reported symptoms, behaviours or observations.	Productive partnership between GPs and people with SCI can benefit from digital diaries for each module with links to the creation of a care plan to enhance sharing of information. This can improve the potential to pick up on any red flags before a crisis. It was recommended that this care plan is linked to annual review on/or about anniversary of onset of SCI.
IV. Individuals	*Innovation Recipients (I)* Individuals who are directly or indirectly receiving the innovation.	The SCI-HMT can help people with SCI to remain healthy and see a brighter future. However, a person with SCI may be overwhelmed by the scale of SCI-HMT content and the requirement for lifelong vigilance. Inclusion of appropriate information addressing the ‘long game’ of SCI is necessary.	The inclusion of lived experience quotes regarding how to self-manage the ‘long game’ for optimal health promotion after SCI is essential. It is recognised that simply “being told what to do” is not helpful.
V. Process	*Innovation Recipients (B2)* Collect information about the priorities, preferences, and needs of recipients to guide implementation and delivery of the innovation.	Four areas for future iterations of the SCI-HMT were identified: (i) sexuality (ii) the taboo nature of bladder and bowel topics for indigenous people, (iii) for SCI-HMT care plans to be compatible with patient management systems, and (iv) leisure as a standalone topic especially the notion of fun.	To ensure longevity, ongoing evaluation of the SCI-HMT by people with SCI, SCI community groups, funders, policy makers and health services is essential to monitor appropriateness of content and identify important gaps which may emerge over time. The digital SCI-HMT can provide regular evidence-based practice updates, with inclusion of links and clinical guidelines, will be far easier to do with a web-based tool/app.

^a^Damschroder, L.J., Reardon, C.M., Widerquist, M.A.O. et al. The updated Consolidated Framework for Implementation Research based on user feedback. *Implementation Sci***17**, 75 (2022). 10.1186/s13012-02

### Innovation

Participants acknowledged that self-management is a major undertaking and very demanding, as one person with SCI said, “*we need to be informed without being terrified and overwhelmed”.* Participants felt the HMT could indeed be adapted, tailored, refined, or reinvented to meet local needs. For example, another person with SCI remarked:

*“Education needs to be from the get-go but in bite sized pieces from all quarters when readiness is most apparent… at all time points*, [not just as a] *a newbie tool or for people with* [long-term impairment]*”* (person with SCI_02).

Therefore, the SCI-HMT had to balance complexity of content while still being accessible and engaging, and required input from both experts in the field and those with lived experience of SCI, for example, a clinical nurse specialist suggested:

*“it’s essential* [the SCI-HMT] *is written by experts in the field as well as with collaboration with people who have had a, you know, the lived experience of SCI”* (healthcare professional_03).

Furthermore, the points of contact with healthcare for a person with SCI can be challenging to navigate and the SCI-HMT has the potential to facilitate a smoother engagement process and improve communication between people with SCI and healthcare services. As a GP suggested:

*“we need a tool like this to link to that pathway model in primary health care*, [the SCI-HMT] *it’s a great tool, something that everyone can read and everyone’s reading the same thing”* (healthcare professional_05).

Participants highlighted that the ability of the SCI-HMT to facilitate effective communication was very much dependent on the delivery format. The idea of digitizing the SCI-HMT garnered equal support from people with SCI and health care professionals, with one participant with SCI deeming it to be “*essential” (*person with SCI_01) and a health professional suggesting a *“digitalized version will be an advantage for most people”* (healthcare professional_02).

### Outer setting

There was strong interest expressed by both people with SCI and healthcare professionals in using the SCI-HMT. The fundamental premise was that knowledge is power and the SCI-HMT would have strong utility in post-acute rehabilitation services, as well as primary care. As a person with SCI said,

“*we need to leave the* [spinal unit] *to return to the community with sufficient knowledge, and to know the value of that knowledge and then need to ensure primary healthcare provider*[s] *are best informed”* (person with SCI_04).

The value of the SCI-HMT in facilitating clear and effective communication and shared decision-making between healthcare professionals and people with SCI was also highlighted, as shown by the remarks of an acute nurse specialist:

*“I think this tool is really helpful for the consumer and the GP to work together to prioritize particular tests that a patient might need and what the regularity of that is”* (healthcare professional_03).

Engaging with SCI peer support networks to promote the SCI-HMT was considered crucial, as one person with SCI emphasized when asked how the SCI-HMT might be best executed in the community, *“…peers, peers and peers”* (person with SCI_01). Furthermore, the layering of content made possible in the digitalized version will allow for the issue of approachability in terms of readiness for change, as another person with SCI said:

“[putting content into a digital format] *is essential and required and there is a need to put summarized content in an App with links to further web-based information… it’s not likely to be accessed otherwise”* (person with SCI_02).

### Inner setting

Participants acknowledged that self-management of health and well-being is substantial and demanding. It was suggested that the scope, tone, and complexity of the SCI-HMT, while necessary, could potentially be resisted by people with SCI if they felt overwhelmed, as one person with SCI described:

*“a manual that is really long and wordy, like, it’s* [a] *health metric… they maybe lack the health literacy to, to consume the content then yes, it would impede their readiness for* [self-management]” (person with SCI_02).

Having support from their GPs was considered essential, and the HMT could enable GP’s, who are under time pressure, to provide more effective health and advice to their patients, as one GP said:

*“We GP’s are time poor, if you realize then when you’re time poor you look quickly to say oh this is a patient tool - how can I best use this?”* (healthcare professional_05).

Furthermore, health professional skills may be best used with the synthesis of self-reported symptoms, behaviors, or observations. A particular strength of a digitized version would be its ability to facilitate more streamlined communication between a person with SCI and their primary healthcare providers developing healthcare plans, as an acute nurse specialist reflected, “*I think that a digitalized version is essential with links to primary healthcare plans”* (healthcare professional_03).

Efficient communication with thorough assessment is essential to ensure serious health issues are not missed, as findings reinforce that the SCI-HMT is an educational tool, not a replacement for healthcare services, as a clinical nurse specialist commented, “*remember, things will go wrong– people end up very sick and in acute care “*(healthcare professional_02).

### Individual

The SCI-HMT has the potential to provide a pathway to a *‘hope for better than now’*, a hope to *‘remain well’* and a hope to *‘be happy’*, as the informant with SCI (04) declared, *“self-management is a long game, if you’re keeping well, you’ve got that possibility of a good life… of happiness”.* Participants with SCI felt the tool needed to be genuine and

*“acknowledge the huge amount of adjustment required, recognizing that dealing with SCI issues is required to survive and live a good life”* (person with SCI_04).

However, there is a risk that an individual is completely overwhelmed by the scale of the SCI-HMT content and the requirement for lifelong vigilance. Careful attention and planning were paid to layering the information accordingly to support self-management as a ‘long game’, which one person with SCI reflected in following:

*“the first 2–3 year* [period] *is probably the toughest to get your head around the learning stuff, because you’ve got to a stage where you’re levelling out, and you’ve kind of made these promises to yourself and then you realize that there’s no quick fix”* (person with SCI_01).

It was decided that this could be achieved by providing concrete examples and anecdotes from people with SCI illustrating that a meaningful, healthy life is possible, and that good health is the bedrock of a good life with SCI.

### Process

There was universal agreement that the SCI-HMT is aspirational and that it has the potential to improve knowledge and understanding for people with SCI, their families, community workers/carers and primary healthcare professionals, as a GP remarked:

“[different groups] *could just read it and realize, ‘Ahh, OK that’s what that means… when you’re doing catheters. That’s what you mean when you’re talking about bladder and bowel function or skin care”* (healthcare professional_04).

Despite the SCI-HMT providing an abundance of information and resources to support self-management, participants identified four gaps: (i) the priority issue of sexuality, including pleasure and identity, as one person with SCI remarked:

“*sexuality is one of the biggest issues that people with SCI often might not speak about that often cause you know it’s awkward for them. So yeah, I think that’s a that’s a serious issue”* (person with SCI_03).

(ii) consideration of the taboo nature of bladder and bowel topics for indigenous people, (iii) urgent need to ensure links for SCI-HMT care plans are compatible with patient management systems, and (iv) exercise and leisure as a standalone topic taking account of effects of physical activity, including impact on mental health and wellbeing but more especially for fun.

To ensure longevity of the SCI-HMT, maintaining a partnership between people with SCI, SCI community groups and both primary and tertiary health services is required for liaison with the relevant professional bodies, care agencies, funders, policy makers and tertiary care settings to ensure ongoing education and promotion of SCI-HMT is maintained. For example, delivery of ongoing training of healthcare professionals to both increase the knowledge base of primary healthcare providers in relation to SCI, and to promote use of the tools and resources through health communities. As a community nurse specialist suggested:

“*improving knowledge in the health community… would require digital links to clinical/health management platforms”* (healthcare professional_02).

In a similar vein, a GP suggested:

“*our common GP body would have continuing education requirements… especially if it’s online, in particular for the rural, rural doctors who you know, might find it hard to get into the city”* (healthcare professional_04).

## Discussion

The successful implementation of evidence-based innovations into practice is dependent on a wide array of dynamic and active contextual factors, including the perspectives of the recipients who are destined to use such innovations. Indeed, the recently updated CFIR has called for innovation recipient perspectives to be a priority when considering contextual factors [10, 11]. Understanding and including the perspectives of those the innovation is being designed to benefit can promote increased equity and validation of recipient populations, and potentially increase the adoption and sustainability of innovations.

In this paper, we have presented research using the recently updated CFIR to guide the collection of innovation recipients’ perspectives (including people with SCI and GPs working in the community) regarding the potential implementation barriers and facilitators of the digital version of the SCI-HMT. Collected data were synthesized to inform actionable findings– tangible ways in which the SCI-HMT could be modified according of the domains of the CFIR (e.g., see Keith et al. [20]). It is important to note that we conducted this research using the original domains of the CFIR [9] prior to Damschroder et al. publishing the updated CFIR [11]. However, in our analysis we were able to align our findings to the revised CFIR domains and constructs, as Damschroder [11] suggests, constructs can “be mapped back to the original CFIR to ensure longitudinal consistency” (p. 13).

One of the most poignant findings from our analyses was the need to ensure the content of the SCI-HMT balanced scientific evidence and clinical expertise with lived experience knowledge. This balance of clinical and experiential knowledge demonstrated genuine regard for lived experience knowledge, and created a more accessible, engaging, useable platform. For example, in the innovation and individual domains, the need to include lived experience quotes was immediately apparent once the perspective of people with SCI was included. It was highlighted that while the SCI-HMT will prove useful to many parties at various stages along the continuum of care following onset of SCI, there will be those individuals that are overwhelmed by the scale of the content. That said, the layering of information facilitated by the digitalized version is intended to provide an ease of navigation through the SCI-HMT and enable a far greater sense of control over personal health and wellbeing. Further, despite concerns regarding e-literacy the digitalized version of the SCI-HMT is seen as imperative for accessibility given the wide geographic diversity and recent COVID pandemic [21]. While there will be people who are challenged by the technology, the universally acceptable use of the internet is seen as less of a barrier than printed material.

The concept of partnership was also apparent within the data analysis focusing on the outer and inner setting domains. In the outer setting domain, our findings emphasized the importance of engaging with SCI community groups, as well as primary and tertiary care providers to maximize uptake at all points in time from the phase of subacute rehabilitation onwards. While the SCI-HMT is intended for use across the continuum of care from post-acute rehabilitation onwards, it may be that certain modules are more relevant at different times, and could serve as key resources during the hand over between acute care, inpatient rehabilitation and community reintegration.

Likewise, findings regarding the inner setting highlighted the necessity of a productive partnership between GPs and individuals with SCI to address the substantial demands of long-term self-management of health and well-being following SCI. Indeed, support is crucial, especially when self-management is the focus. This is particularly so in individuals living with complex disability following survival after illness or injury [22], where health literacy has been found to be a primary determinant of successful health and wellbeing outcomes [23]. For people with SCI, this tool potentially holds the most appeal when an individual is ready and has strong partnerships and supportive communication. This can enable potential red flags to be recognized earlier allowing timely intervention to avert health crises, promoting individual well-being, and reducing unnecessary demands on health services.

While the SCI-HMT is an educational tool and not meant to replace health services, findings suggest the current structure would lead nicely to having the conversation with a range of likely support people, including SCI peers, friends and family, GP, community nurses, carers or via on-line support services. The findings within the process domain underscored the importance of ongoing partnership between innovation implementers and a broad array of innovation recipients (e.g., individuals with SCI, healthcare professionals, family, funding agencies and policy-makers). This emphasis on partnership also addresses recent discussions regarding equity and the CFIR. For example, Damschroder et al. [11] suggests that innovation recipients are too often not included in the CFIR process, as the CFIR is primarily seen as a tool intended “to collect data from individuals who have power and/or influence over implementation outcomes” (p. 5).

Finally, we feel that our inclusion of innovation recipients’ perspectives presented in this article begins to address the notion of equity in implementation, whereby the inclusion of recipient perspectives in research using the CFIR both validates, and increases, the likelihood of sustainable adoption of evidence-based innovations, such as the SCI-HMT. We have used the CFIR in a pragmatic way with an emphasis on meaningful engagement between the innovation recipients and the research team, heeding the call from Damschroder et al. [11], who recently argued for researchers to use the CFIR to collect data from innovation recipients. Adopting this approach enabled us to give voice to innovation recipient perspectives and subsequently ensure that the tone, scope, content and presentation of the SCI-HMT balanced the needs of innovation recipients alongside the provision of evidence-based clinical information.

Our research is not without limitations. While our study was successful in identifying a number of potential barriers and facilitators to the implementation of the SCI-HMT, we did not test any implementation strategies to impact determinants, mechanisms, or outcomes. This will be the focus of future research on this project, which will investigate the impact of implementation strategies on outcomes. Focus will be given to the context-mechanism configurations which give rise to particular outcomes for different groups in certain circumstances [7, 24]. A second potential concern is the relatively small sample size of participants that may not allow for saturation and generalizability of the findings. However, both the significant impact of secondary health complications for people with SCI and the desire for a health maintenance tool have been established in Australia [2, 4]. The aim our study reported in this article was to achieve context-specific knowledge of a small sample that shares a particular mutual experience and represents a perspective, rather than a population [25, 26]. We feel our findings can stimulate discussion and debate regarding participant-informed approaches to implementation of the SCI-HMT, which can then be subject to larger-sample studies to determine their generalisability, that is, their external validity. Notably, future research could examine the interaction between certain demographic differences (e.g., gender) of people with SCI and potential barriers and facilitators to the implementation of the SCI-HMT. Future research could also include the perspectives of other allied health professionals working in the community, such as occupational therapists. Lastly, while our research gave significant priority to recipient viewpoints, research in this space would benefit for ensuring innovation recipients are engaged as genuine partners throughout the entire research process from conceptualization to implementation.

## Conclusion

Employing the CFIR provided an effective, systematic method for identifying recipient perspectives regarding the implementation of a digital health maintenance tool for people living with SCI. Findings emphasized the need to balance clinical and lived experience perspectives when designing an implementation strategy and facilitating strong partnerships with necessary stakeholders to maximise the uptake of SCI-HMT into practice. Ongoing testing will monitor the uptake and implementation of this innovation, specifically focusing on how the SCI-HMT works for different users, in different contexts, at different stages and times of the rehabilitation journey.

### Electronic supplementary material

Below is the link to the electronic supplementary material.

Supplementary Material 1

## Data Availability

The datasets supporting the conclusions of this article are available available upon request and with permission gained from the project Steering Committee.

## Abbreviations

SCI: spinal cord injury
SCI: HMT-Spinal Cord Injury Health Maintenance Tool
CFIR: Consolidated Framework for Implementation Research
