# Supplementary material for: Using the consolidated Framework for Implementation Research to integrate innovation recipients’ perspectives into the implementation of a digital version of the spinal cord injury health maintenance tool: a qualitative analysis
Source: BMC Health Serv Res. 2024 Mar 28;24:390. doi: 10.1186/s12913-024-10847-x (PMC10976821; doi:10.1186/s12913-024-10847-x)
Supplement: Supplementary file 1 — Supplementary Material 1 [file 12913_2024_10847_MOESM1_ESM.docx]

**Supplementary File 1:** Semi-structured interview guide developed according to the original 2009 CFIR domains^1^.

I INNOVATION: SCI HEALTH MAINTENANCE TOOL

1. Do you think the HMT has the potential to assist with the pathway through the maze?
2. How might self-management be enhanced?
3. What are the likely points of contact?
4. How transferable do you believe the tool contents might be?
5. How might the digitalized version assist transfer information across those points of contact?

II OUTER SETTING

1. Thinking here about one’s culture how best might this tool be communicated within the NSW community?
2. How aspirational is it to imagine this tool will reach broader SCI group in NSW?
3. What might SCI peers have to offer?

III INNER SETTING

1. Based on the idea that all human beings are members of a single community how ready do you believe individuals with SCI are for a self-management tool?
2. What personal needs might be foremost on the minds of individuals and their GP’s?

IV INDIVIDUALS

1. How might personal health beliefs impact on the tool’s utility?
2. Might the tool facilitate readiness for change?
3. Might the tool impede readiness for change?

V IMPLEMENTATION PROCESS

1. How do we ensure individuals with SCI identify with the process?
2. Who?
3. How?
4. When?
5. How much of an advantage do you think for you from your experience, the digitalised version might be?
6. Barriers to the digitalized tool?
7. What do you believe a digitalized tool might provide for individuals with SCI living in the community in terms of relative advantage?
8. Process + Time and Money/Costs: What would you consider the best approach to executing it into the SCI community?
9. How best might we seek reflection on its use in the broader sector?
10. Uptake/incentives?
11. How best might we evaluate the tool in terms of success or failure?

VI GAPS: WHAT HAVE WE MISSED?

1. Are there areas you think we might have missed or could improve the tool?

^1^ Damschroder, L. J., Aron, D. C., Keith, R. E., Kirsh, S. R., Alexander, J. A., & Lowery, J. C. (2009). Fostering implementation of health services research findings into practice: a consolidated framework for advancing implementation science. *Implementation Science, 4*(1), 50. doi:10.1186/1748-5908-4-50
